# Disorder Driven Anisotropic Nonlinear Hall Effect in the Kagome Metal YbCo_6_Ge_6_ at Room Temperature

**DOI:** 10.1002/advs.77434

**Published:** 2026-08-27

**Authors:** Qurat Ul Ain, Weiyao Zhao, Thanh Tung Huynh, Yuefeng Yin, Fang Tang, Yong Fang, Junlin Yan, Jacek Jasieniak, Timothy Petersen, Yang Liu, Nikhil V. Medhekar, Julie Karel

**Affiliations:** ^1^ Department of Materials Science & Engineering Monash University Clayton VIC Australia; ^2^ Jiangsu Laboratory of Advanced Functional Materials School of Electronic and Information Engineering Suzhou University of Technology Changshu China; ^3^ Monash Centre for Electron Microscopy (MCEM) Monash University Clayton VIC Australia; ^4^ School of Physics and Astronomy Monash University Clayton VIC Australia

**Keywords:** berry curvature, kagome metal, nonlinear hall effect, skew scattering

## Abstract

We show that the kagome metal YbCo_6_Ge_6_ displays a remarkable phenomenon: its intrinsic structural disorder and short‐range correlated motifs, rather than diminishing performance, constitute the primary mechanism for a robust and anisotropic nonlinear Hall effect (NLHE). This effect is sustained across a wide temperature range from 2 to 350 K, achieving a nonlinear responsivity of up to ∼4.5 × 10^−3^ m/V at 70 K, higher than known disordered and engineered quantum heterostructures. Extrinsic scattering mechanisms, modulated by structural disorder variations in the kagome lattice, primarily govern the nonlinear Hall response. Yet, a nearly temperature‐independent intrinsic Berry curvature dipole signal persists, demonstrating that quantum geometry remains significant despite the presence of disorder. These findings challenge the prevailing view that structural disorder in three‐dimensional quantum materials are detrimental and should be minimized. Instead, such correlated disorder is shown to be functional assets, establishing a new paradigm in materials design where short‐range motifs, rather than perfect crystallinity, enable room‐temperature nonlinear Hall functionality for advanced rectification and detection technologies.

## Graphical abstract

The intrinsic structural disorder and short‐range correlated motifs in the kagome metal YbCo_6_Ge_6_ constitute the primary mechanism for a robust and anisotropic nonlinear Hall effect (NLHE). This effect is sustained across a wide temperature range, achieving a nonlinear responsivity of up to ∼4.5 × 10^−3^ m/V at 70 K, higher than known disordered and engineered quantum heterostructures.

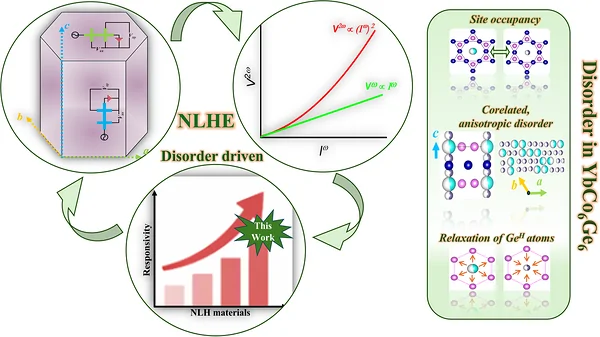

## 1 Introduction

The Hall effect has long served as a fundamental tool for investigating charge transport and electronic structure in condensed matter systems. Its practical application is limited by the need for magnetic materials and/ or external magnetic fields, which restricts the miniaturization and integration of Hall‐based rectifiers, detectors and energy‐harvesting devices in modern electronics [1, 2]. The nonlinear Hall effect (NLHE) discovered recently, overcomes this limitation by enabling transverse voltage generation in nonmagnetic systems that preserve time‐reversal symmetry but lack inversion symmetry [3, 4]. As a second‐order response, an alternating electric field induces a transverse voltage that scales quadratically with the applied field, allowing an alternating current to generate both second‐harmonic (2*ω*) and rectified dc Hall signals (*V^RDC^*) without magnetic fields (***B*)** [4]. This development enables electrical control of Hall responses, with implications for high‐frequency detection, wireless signal processing and on‐chip energy conversion [5, 6, 7, 8, 9, 10].

Despite the promise of NLHE, its implementation in practical devices remains challenging. Early experimental demonstrations have focused on quantum materials such as topological semimetals [11, 12, 13, 14, 15, 16, 17], engineered heterostructures [13, 18, 19], and twisted bilayer van der Waals systems [20, 21, 22]. These materials have advanced the understanding of the underlying physics, yet they present limitations: many operate only at cryogenic temperatures, require atomically precise fabrication, or depend on fragile electronic structures that are difficult to scale [11, 12, 13, 14, 15, 16, 17, 18, 19, 20, 21, 22]. Additionally, the mechanisms underlying the intrinsic NLHE, for instance, the Berry curvature dipole (BCD, **D**) and quantum metric dipole (QMD) in strongly correlated and/or magnetic topological materials, are often sensitive to disorder and structural imperfections [11, 12, 13, 14, 15, 16, 17, 23, 24, 25, 26]. This raises a fundamental question for device engineering regarding whether disorder must always be minimized to observe the NLHE, or if it can also be harnessed as a functional component.

Kagome materials offer a promising platform to address this question, as their geometry and electronic structure inherently enhance nonlinear transport. The kagome lattice composed of corner‐sharing triangles, supports a complex interplay of Dirac crossings (DC), flatbands (FB), and van Hove singularities (vHS), all amplified by spin‐orbit coupling (SOC) [26, 27, 28]. These features produce significant variations in Berry curvature and promote strong electronic correlations, making them highly sensitive to symmetry breaking and external perturbations [26, 27]. Moreover, kagome materials inherently exhibit geometrical frustration, which supports disorder‐driven extrinsic contributions to the NLHE, such as skew scattering and side jump due to naturally increased scattering asymmetries [26, 28]. Taking account of these structural modifications, perhaps disorder can be leveraged as a design parameter to generate robust nonlinear responses rather than being viewed solely as a perturbation to minimize.

The Kagome metal, YbCo_6_Ge_6_ a newly synthesized member of the *RT*_6_ *X*_6_ (*R* = rare earth; *T* = transition metal; *X* = Ge, Sn) family, exemplifies this approach. Unlike idealized model systems, YbCo_6_Ge_6_ exhibits occupational and structural disorder as an intrinsic aspect of its crystal structure and not an extrinsic imperfection [29, 30, 31].YbCo_6_Ge_6_ undergoes a structural transition from a high‐symmetry hexagonal phase *P6/mmm* to a lower‐symmetry *P6_3_/m* phase at ∼95 K reminiscent of charge density wave (CDW) formation [30, 31]. Intriguingly, first‐principles calculations reveal the presence of higher‐order saddle points (HOSPs) near the Fermi level, which generate extended flatbands (FBs) and could drive electronic instabilities in YbCo_6_Ge_6_ [32]. These attributes position YbCo_6_Ge_6_ at a unique intersection of frustration, disorder, and electronic complexity, where scattering‐driven transport effects are naturally enhanced. In this study, we show that the disorder in YbCo_6_Ge_6_ does not suppress the nonlinear Hall response, instead it enables it. A robust, anisotropic NLHE is observed from cryogenic temperatures to 350 K, surpassing room temperature. The scaling behaviour indicates that the effect is predominantly extrinsic, arising from disorder‐mediated scattering with intrinsic BCD as a secondary contribution. These results establish YbCo_6_Ge_6_ as a model system in which disorder acts as an active mechanism, enabling room‐temperature nonlinear Hall functionality without the need for magnetic fields or complex materials engineering.

## 2 Results

**FIGURE 1:**
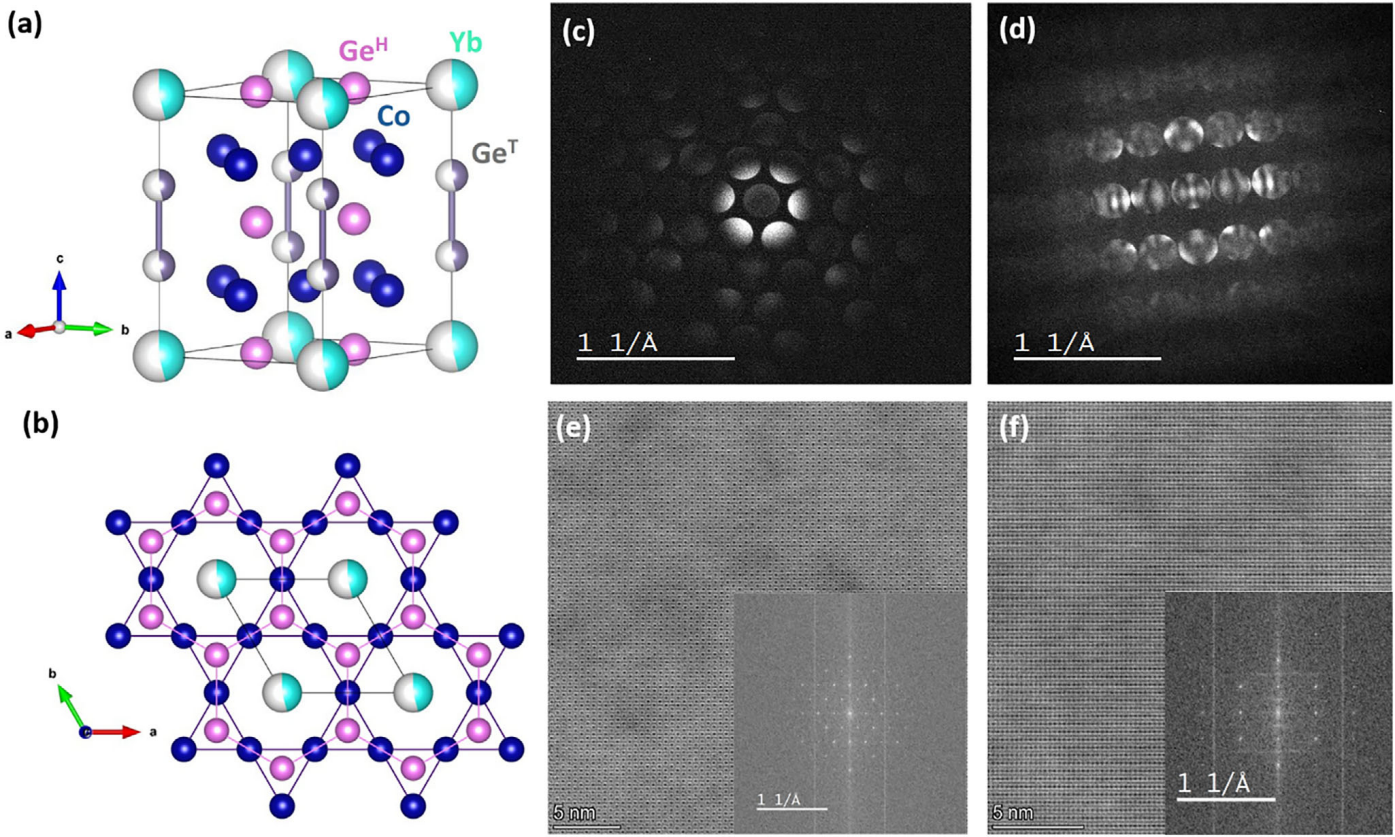
Structural and electronic characterization of YbCo_6_Ge_6_ single crystals. (a, b) Crystal structure of pristine (*P6/mmm* phase) YbCo_6_Ge_6_, an averaged structure of disordered HfFe_6_Ge_6_ which exhibits partial occupancy at Yb and Ge^T^ sites, viewed along the (a) *c*‐axis [0001] and (b) *ab*‐plane. Ge^H^ and Ge^T^ corresponds to hexagonal and trigonal sites of Ge atoms. (c, d) Room temperature convergent beam electron diffraction (CBED) patterns acquired along the (c) [0001] zone axis and (d) an orthogonal direction from the <$11\bar{2}0$> family. (e, f) High‐magnification annular bright field (ABF) images recorded along the (e) [0001] and (f) orthogonal <$11\bar{2}0$> directions. Insets show the corresponding power spectra, confirming the crystalline quality and spatial frequencies consistent with the CBED patterns. TEM experiments were conducted on the sample crystal piece from which Hall bar devices S9 and S10 were prepared for transport studies.

Figure 1 represents the crystal structure and electronic band structure of YbCo_6_Ge_6_. At room temperature, YbCo_6_Ge_6_ adopts the disordered HfFe_6_Ge_6_‐type structure (space group, *P6*/*mmm*), integrating the structural motifs of the YCo_6_Ge_6_ and CoSn structures without long‐range order in the Yb and Ge atoms along the *c*‐axis, as shown in Figure 1a. More specifically, the structure consists of ideal Co‐kagome layers in the *ab*‐plane (Figure 1b). There are two distinct Ge sites; the Ge (Ge^T^) atom at the trigonal site forms a dimer along the *c*‐axis, while the Ge^H^ at the hexagonal sites form the honeycomb layers with Yb atoms occupying their central position [30, 33, 34]. The room temperature crystal structure was investigated using convergent beam electron diffraction (CBED) along two principal zone axes: the *c*‐axis [0001] (Figure 1c) and an orthogonal direction from the <$11\bar{2}0$> family (Figure 1d). The CBED patterns exhibited no evidence of diffuse scattering or Gjönnes‐Moodie (G‐M) extinction lines, ruling out the presence of screw axes or glide planes. Based on kinematical analysis, the pattern in Figure 1c allowed us to distinguish between the *P6/mmm* and *P6_3_/m* phases, confirming that the crystal bulk adopts the pristine high‐temperature *P6/mmm* structure. The pronounced Bragg disk contrast arises from strong non‐kinematical scattering due to the substantial matrix thickness (∼50 nm) from which the nano‐beam CBED patterns were acquired. High‐magnification annular bright field (ABF) images (Figure 1e,f) further confirm the high crystalline quality of the crystal, which is largely free of stacking faults. The corresponding power spectra (insets) display spatial frequencies consistent with the CBED disk centers shown in Figure 1c,d. Minor diffuse contrast is attributed to surface roughening caused by Gallium ion milling. Overall, the TEM analysis reveals a high‐quality bulk crystal with only sparse, minor defects (see Note S1 for details).

**FIGURE 2:**
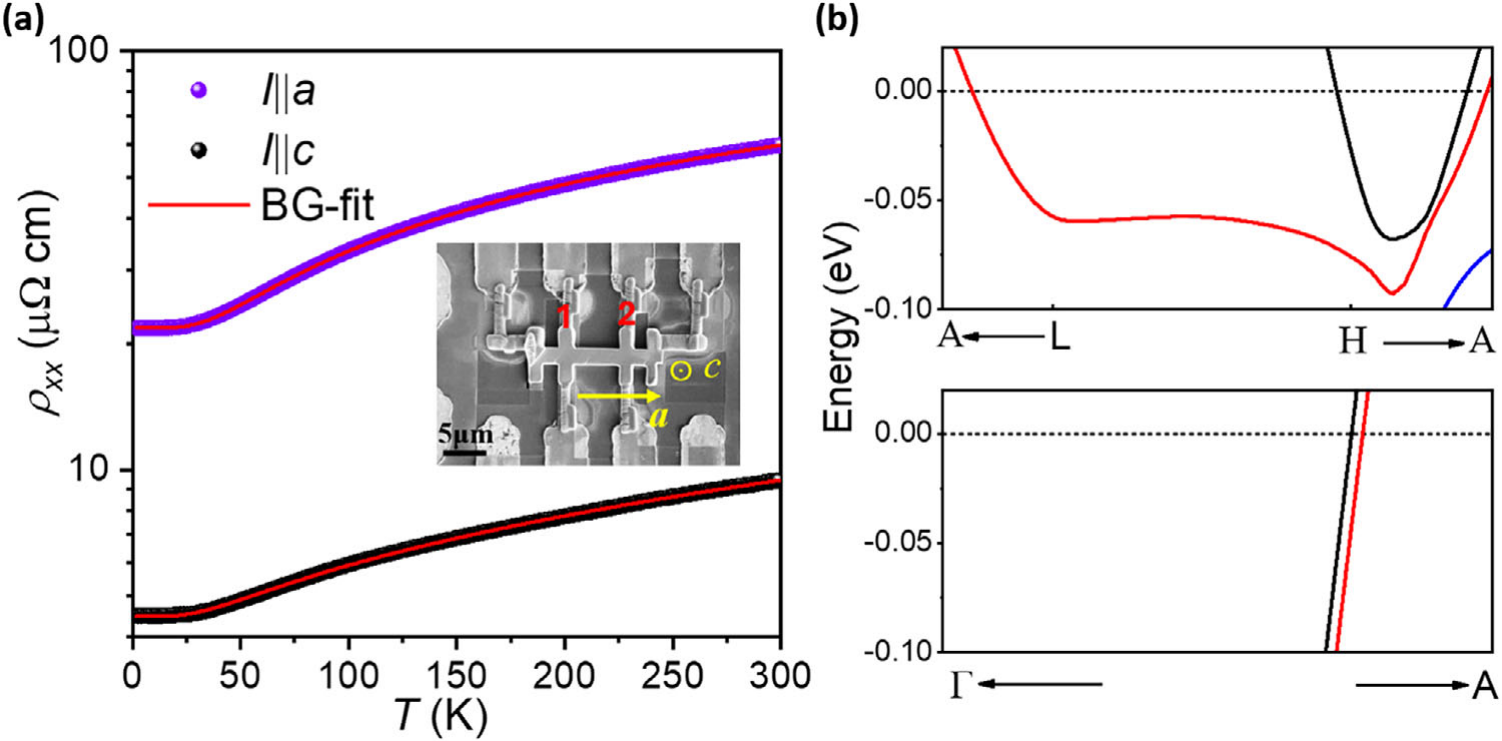
Charge transport anisotropy in YbCo_6_Ge_6_. (a) Temperature‐dependent longitudinal resistivity (*ρ_xx_*) measured with the applied current parallel to the *c*‐axis (black spheres) and *a*‐axis (violet). Red solid lines are the fits to SI Equation (1). Inset: Secondary electron (SE) microscope image of a representative focused ion beam (FIB)‐milled Hall bar device (device S7) oriented along the crystallographic *a*‐axis. The longitudinal resistivity is measured between voltage leads 1 and 2. Multiple Hall bars with varying dimensions were fabricated and measured; the data presented in the main text are for devices S9 (*I*||*c*) and S10 (*I*||*a*). Device S9 has dimensions of 15.6 µm (length) × 1.2 µm (width) × 1.2 µm (thickness), with a voltage‐lead spacing of 7.5 µm. Device S10 measures 11.6 µm × 1.2 µm × 1.2 µm, with a voltage‐lead spacing of 7 µm. The SE image in the inset shows device S7, which has a width of ∼1.78 µm and is otherwise geometrically comparable to S9. (b) Typical energy dispersion of electronic bands near the Fermi level along the *k_x_* = *k_y_* direction (top panel) and the *k_z_* direction (bottom panel), highlighting the anisotropic band structure underlying the transport behavior for *P*6/*mmm* phase.

The charge transport in YbCo_6_Ge_6_ was also investigated. Figure 2a depicts the temperature dependence of longitudinal resistivity (*ρ_xx_*) for the current flowing parallel (*I||a*) and perpendicular (*I||c*) to the Kagome plane (*ab*). For both the orientations, *ρ_xx_* shows metallic behavior. No anomalies associated with charge density wave (CDW) formation or magnetic ordering were observed, in agreement with magnetometry measurements (see Note S2). These results confirm the paramagnetic character of YbCo_6_Ge_6_. The red solid lines in Figure 2a are the fits to Bloch–Grüneisen (BG) model over the complete temperature region (See Note S3). The strong anisotropy (ρ_*a*_/ρ_*c*_ = ∼4.85 at 2 K) in electrical resistivity observed can be associated with the nearly flatband (FB) generated by the HOSP ζ_1_ at *L* across the *L*‐*H* direction (*k_x_—k_y_* plane) near the Fermi level at −0.058 eV (see Figure 2b top panel and Note S10 for detailed band structure). The ζ_1_‐associated band exhibits flatter dispersions near the Fermi level, contributing to a pronounced density of states (DOS) peak that contributes mainly to the electronic transport properties of YbCo_6_Ge_6_ [32]. Since HOSP and FBs correspond to larger effective masses and quenched kinetic energies, carrier movement within the kagome plane is suppressed, leading to higher resistivity (*I||a*). In contrast, the steeper band dispersion along the *Γ*‐*A* direction (*k_z_*) results in a lower effective mass and reduced resistivity out of the Kagome plane (*I*||*c*), see Figure 2b bottom panel. The calculated electronic and phonon band structures for *P6mmm* phase are presented in SI Fig. and Note 10. The Hall effect measurements indicate electron‐dominated conduction, with anisotropic carrier mobilities consistent with the material's electronic structure and longitudinal resistivities (see Note S4 for details). While a linear Hall effect and anisotropic mobilities occurs routinely in conventional, weakly correlated metals with structurally asymmetrical effective masses, their manifestation in YbCo_6_Ge_6_ carries deeper implication tied to the material's underlying kagome band topology. In this system, charge transport is heavily dictated by highly directional electronic features (e.g. FBs, HOSP, DP) native to the kagome sublattice geometry. These unique features localize carriers within specific crystallographic planes, amplifying local electronic correlation effects thus giving rise to distinct direction‐dependent scattering channels observed in our data.

**FIGURE 3:**
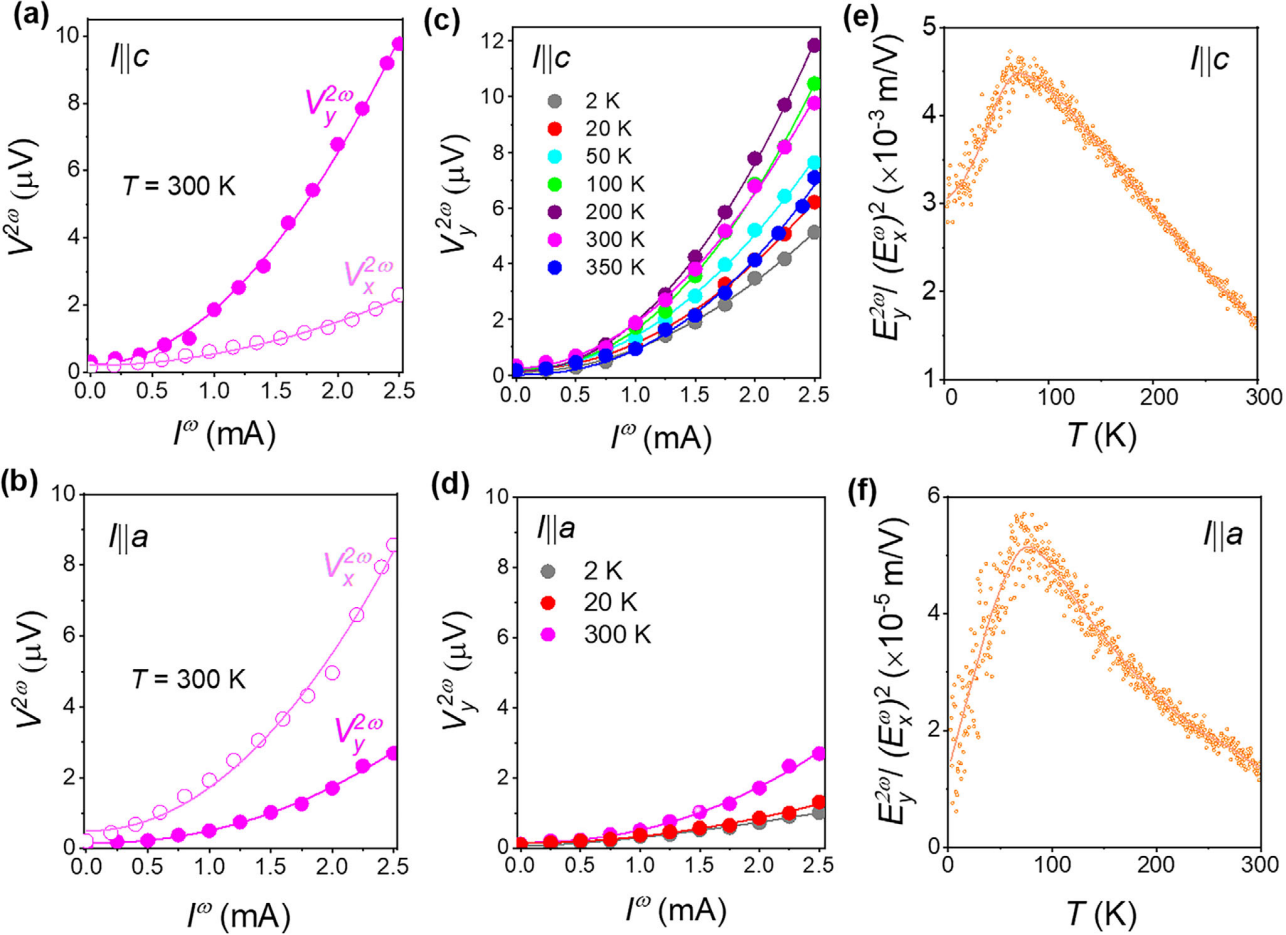
Nonlinear electronic transport in YbCo_6_Ge_6_. (a, b) Second harmonic longitudinal *V_x_^2ω^* (open circles) and Hall voltage *V_y_^2ω^* (solid circles) as a function of driving current *I^ω^* at room temperature for *I*||*c* (a) and *I*||*a* (b). Circles are the experimental data points while the solid lines are the quadratic fits. (c, d) Second harmonic Hall voltage *V_y_^2ω^* as a function of driving current *I^ω^* at various temperatures for current flowing out of plane (*I*||*c*) (c) and current flowing in plane (*I*||*a*) (d). (e, f) Temperature dependence of NLHE strength $\frac{E_{y}^{2\omega }}{{\left(E_{x}^{\omega }\right)}^{2}}$ known as responsivity for *I*||*c* (e) and *I*||*a* determined from temperature dependence of *V_y_^2ω^* shown in Figure S7c. (f). The orange solid line in (e, f) is a guide to eye obtained by adjacent‐averaging of raw data. The measurements were performed on Hall bar device S9 for *I*||*c* and S10 for *I*||*a*.

The nonlinear electronic transport properties of YbCo_6_Ge_6_ with current applied along both the in‐plane (*I*∥*a*) and out‐of‐plane (*I*∥*c*) directions were studied. A finite second harmonic voltage was observed for both orientations under an alternating current excitation *I^ω^*, exhibiting a quadratic dependence on the excitation amplitude (*V^2ω^* ∝ (*I^ω^*)^2^), which confirms the second‐order nature of the nonlinear response. The possibilities of NLHE associated with extrinsic effects such as the contact junction effect, asymmetric sample shape, thermoelectric and Joule heating effect were eliminated (see Note S5). At room temperature, the transverse (Hall) second harmonic signal *V_y_^2ω^* was substantially larger for currents applied along the *c*‐axis (*I*∥*c*) than for those along the *a*‐axis (*I*∥*a*), as shown by the solid circles in Figure 3a,b, revealing a pronounced anisotropy. Notably, for an in‐plane current (*I*∥*a*), the longitudinal second harmonic voltage *V_x_^2ω^* is larger than the transverse component *V_y_^2ω^*, indicating longitudinal voltage dominance in this configuration. Conversely, for out‐of‐plane current (*I*∥*c*), the Hall voltage *V_y_^2ω^* exceeded the longitudinal component *V_x_^2ω^*, revealing the Hall voltage dominance. Together, these observations point to distinct underlying nonlinear transport mechanisms depending on the direction of the excitation current, that are possibly related to correlated disorder in this material, spanning different length scales along the two orientations (discussed later in detail).

The simultaneous emergence of both *V_y_^2ω^* and *V_x_^2ω^* suggests contributions from both intrinsic and extrinsic mechanisms to the nonlinear electronic transport. For *I*∥*c* (Figure 3c), the NLHE persists across a remarkably wide temperature range from 2 to 350 K, displaying a non‐monotonic temperature dependence. The second‐harmonic response *V_y_^2ω^* increases gradually with temperature, forming a broad peak above 100 K and reaching a maximum around 200 K (see Note S6, Figure S5a). The subsequent reduction at higher temperatures coincides with the Debye temperature (Θ_D_ = 222.1 K, Table S1), suggesting that the onset of the high‐temperature suppression is driven by increased electron‐phonon scattering as additional lattice vibration modes become thermally activated. Despite this moderate decline above 200 K, the NLHE remains robust up to room temperature, with a sizeable transverse voltage of ∼10 µV at 2.5 mA. This translates to a nonlinear Hall angle of ∼77° at 300 K, highlighting the material's significant potential for practical device applications. In contrast, for *I*∥*a* (Figure 3d), the transverse nonlinear Hall voltage *V_y_^2ω^* remains weak across the entire measured temperature range, with a minimal Hall angle of only ∼6° at 300 K. Meanwhile, the longitudinal component *V_x_^2ω^* for this orientation exhibits a distinct temperature profile, peaking around 100 K before gradually decreasing (see Note S6 and Figure S5b).

A key figure of merit in NLHE systems is the NLHE responsivity, which quantifies the strength of nonlinear response. As shown in Figure 3e,f for current flowing through two orientations (*I*∥*c* and *I*∥*a*), responsivity is defined as $\frac{E_{y}^{2\omega }}{{\left(E_{x}^{\omega }\right)}^{2}}$, where $E_{y}^{2\omega }$ and $E_{x}^{\omega }$ are the nonlinear transverse and the longitudinal electric fields, respectively. They are given by $E_{y}^{2\omega }=\frac{V_{y}^{2\omega }}{d}$ and $E_{x}^{\omega }=\frac{V_{x}^{\omega }}{L}$, with *L* and *d* being the length and width of channels as function of temperature. The peaks in $\frac{E_{y}^{2\omega }}{{\left(E_{x}^{\omega }\right)}^{2}}$ indicate the maximized intrinsic BCD contribution for time‐reversal symmetric systems (since |**D|** $\propto \frac{E_{y}^{2\omega }}{{\left(E_{x}^{\omega }\right)}^{2}}$). The maximum in $\frac{E_{y}^{2\omega }}{{\left(E_{x}^{\omega }\right)}^{2}}$ appears around ∼70 K (Figure 3e) for *I*∥*c*, corresponding to largest BCD and yielding a NLH angle of ∼88.5°. This enhancement may be linked with the possible low‐symmetry phases due to lattice distortions, which can induce nonlinear transport, as supported by our phonon band structure DFT calculations (SI Note 10) and consistent with Ref. [32]. Because the second harmonic charge transport is highly sensitive to symmetry breaking, it can provide a more direct probe of low symmetry structural transitions than linear transport. Previous studies reported the CDW transition near ∼95 K in ref. [30]. and 320 K in ref. [29], the wide variation reflects differences in disorder strength, which is known to suppress the transition temperature. Taking account of this, the maxima observed in Figure 3(e) might be associated with the low‐symmetry *P6_3_/m* phase. However, the lower temperature observed in our measurements therefore suggests a higher degree of disorder in our samples. Interestingly, for *I*∥*a* a peak appears at approximately the same temperature (Figure 3f), despite the BCD being strongly constrained along the *c* axis. This feature likely reflects the same underlying lattice distortions, although the resulting nonlinear transverse response is much weaker due to the reduced Berry curvature asymmetry in the *ab* plane. The NLHE in YbCo_6_Ge_6_, therefore, is driven by symmetry breaking resulting in structural modifications due to the correlated disorder in this material. These results have been reproduced in other Hall bar devices, see Note S11 for details.

**FIGURE 4:**
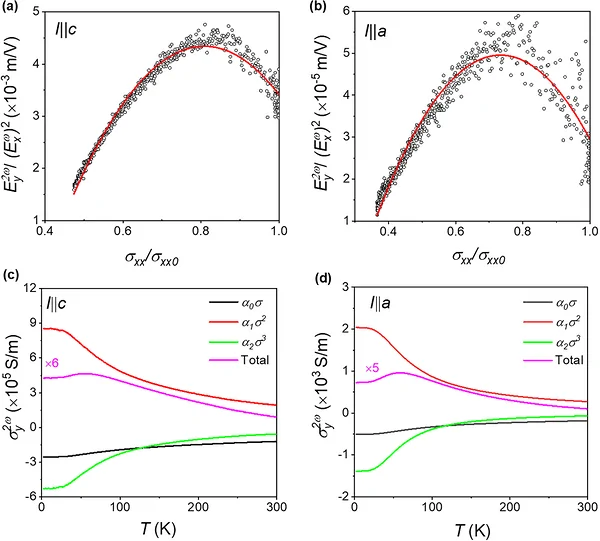
Scaling analysis for YbCo_6_Ge_6_. (a, b) Parabolic fitting of $\frac{E_{y}^{2\omega }}{{\left(E_{x}^{\omega }\right)}^{2}}$ vs. $\frac{\sigma_{xx}}{\sigma_{0}}$ for *I*∥*c* (a) and *I*∥*a* (b). (c, d) Weights of terms in Equation (3) calculated for *I*∥*c* (c) and *I*∥*a* (d) contributing to $\sigma_{y}^{2\omega }$. In (c, d) *σ* is actually representing *σ*_*xx*_.

To identify the intrinsic and extrinsic contributions, we carried out scaling analysis of $\frac{E_{y}^{2\omega }}{{\left(E_{x}^{\omega }\right)}^{2}}$ = $\frac{\sigma_{y}^{2\omega }}{\sigma_{xx}}$, vs. normalized longitudinal conductivity $\frac{\sigma_{xx}}{\sigma_{0}}$, where *σ*_*0*_ = *σ* (2 K) and $\sigma_{y}^{2\omega }$ is second harmonic conductivity tensor as defined in Equation (1) (see Figure 4a,b) for the observed nonlinear Hall signal [22, 35, 36, 37]. The intrinsic component associated with the BCD (**D**) is relatively straightforward to identify due to its dependence on transport relaxation time and leads only to a transverse nonlinear voltage (*V_y_^2ω^*) [12, 38]. In contrast, the extrinsic contributions arising mainly from side jump and skew scattering are relatively complicated to separate and depend sensitively on the type, strength and size (spatial distribution) of disorder [9, 35, 37]. According to theory, $\frac{E_{y}^{2\omega }}{{\left(E_{x}^{\omega }\right)}^{2}}$ can be scaled as [22, 37, 39, 40]:

(1) $$\frac{E_{y}^{2\omega }}{{\left(E_{x}^{\omega }\right)}^{2}}=\frac{\sigma_{y}^{2\omega }}{\sigma_{xx}}=\alpha_{0}+\alpha_{1}\left(\frac{\sigma_{xx}}{\sigma_{0}}\right)+\alpha_{2}{\left(\frac{\sigma_{xx}}{\sigma_{0}}\right)}^{2}$$

where *α*_*i*_ (*i* = 0, 1, 2) indicate the mixed contributions from intrinsic, extrinsic side jump, and extrinsic skew scattering which is further categorised into Gaussian and non‐Gaussian skew scattering mechanisms. Fitting parameters in Equation (1) are defined as; $\alpha_{0}=C^{INT}+C_{1}^{SJ}+C_{11}^{SK,1}$; $\alpha_{1}=C_{01}^{SK,1}-2C_{11}^{SK,1}+C_{0}^{SJ}-C_{1}^{SJ}$ and $\alpha_{2}=C^{SK,2}\;\sigma_{0}+C_{00}^{SK,1}+C_{11}^{SK,1}-C_{01}^{SK,1}$, where *C^INT^* indicate the intrinsic mechanism, *SJ* denotes side jump and *SK* is used for skew scattering, *SK*,1 and *SK*, 2 indicate the Gaussian and non‐Gaussian skew scattering, the subscript 0 and 1 indicate the static (impurity, defects) and dynamic (phonon) scatterings respectively.

Fitting Equation (1) to the experimental data $\frac{E_{y}^{2\omega }}{{\left(E_{x}^{\omega }\right)}^{2}}$ vs. $\frac{\sigma_{xx}}{\sigma_{0}}$ as shown in Figure 4a,b for the whole temperature range measured from 2 to 300 K, resulted in non‐zero values of all the fitting *α*_*i*_ parameters This indicates that the measured NLHE has all the three components corresponding to the three terms on the right of Equation (1). This is consistent with the expectation of both intrinsic and extrinsic mechanisms contributing to observed NLHE in disordered YbCo_6_Ge_6_, as inferred from the simultaneous observation of *V_y_^2ω^* and *V_x_^2ω^* in Figure 3a–d. The fitting parameters extracted are tabulated in Table S2. The opposite signs of all three parameters indicate the competing nature of the three mechanisms. Figure S9 presents the temperature evolution of the individual terms and their relative weightage contributions to the responsivity $\frac{E_{y}^{2\omega }}{{\left(E_{x}^{\omega }\right)}^{2}}$.

In Figure 4c,d, we decompose the total nonlinear conductivity ($\sigma_{y}^{2\omega }$), into three distinct contributions: 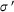, * σ* ^*′′*^, and  *σ* ^*′′′*^ corresponding to increasing powers of the longitudinal conductivity as *α*_*0*_ *σ*_*xx*_, $\alpha_{1}\sigma_{xx}^{2}$ and $\alpha_{2}\sigma_{xx}^{3}$ respectively, for current flowing along *I*∥*c* and *I*∥*a*. These components represent different physical mechanisms underlying the nonlinear Hall effect (NLHE) in YbCo_6_Ge_6_. Notably, the total $\sigma_{y}^{2\omega }$, obtained from this fitting procedure precisely matches the experimental second‐harmonic voltage $V_{y}^{2\omega }$, as validated in Figure S7d. For both current configurations, *I*∥*c* and *I*∥*a*, 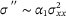, emerges as the dominant contribution across the entire temperature range. This term originates from impurity and phonon‐induced side‐jump and skew‐scattering mechanisms, highlighting the crucial role of extrinsic scattering processes in generating the nonlinear transverse voltage. At 2 K, where *σ*_*xx*_, approaches its residual value *σ*_*xx*0_, and lattice vibrations are frozen out, the NLHE arises exclusively from static scattering from impurities and defects. Under these conditions, 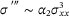, attributed to Gaussian and non‐Gaussian skew scattering constitutes the second‐largest contribution. Since phonon‐mediated processes are absent at low temperatures, both  *σ* ^*′′*^ and  *σ* ^*′′′*^ in this regime reflect purely static disorder effects. This interpretation is corroborated by the material's low residual resistivity ratio (RRR, see Table S1), confirming the presence of substantial disorder [29, 34].

Static scattering alone cannot fully account for the observed NLHE. The *α*_*0*_ *σ*_*xx*_ term, though smaller in magnitude, exhibits a nonzero contribution that persists even at the lowest temperatures. This term encompasses both intrinsic band‐structure effects and dynamic scattering processes. In the low‐temperature limit, dynamic (phonon) scattering is negligible, leaving the intrinsic mechanism specifically, the Berry‐curvature dipole (BCD, **D**) in this time‐reversal‐symmetric system as the essential component of 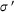. Thus, at *T* → 0 K (2 K in our experiment), the NLHE in YbCo_6_Ge_6_ arises from a combination of static scattering (side jump and skew scattering) and the intrinsic BCD contribution. As temperature increases, both  *σ* ^*′′*^ and  *σ* ^*′* *′′*^, decrease monotonically, signalling a crossover from static to dynamic scattering dominance. This decline in static scattering reflects energy dissipation and decoherence introduced by multiple scattering events with thermally activated phonons. Notably, the  *σ* ^*′* ^term remains nearly temperature‐independent throughout, indicating that the BCD contribution, while secondary in magnitude, is remarkably robust and indispensable for a complete description of the experimental signal.

## 3 Discussion

The observation of the nonlinear Hall effect (NLHE) in YbCo_6_Ge_6_ at room temperature necessitates inversion symmetry breaking, yet our convergent beam electron diffraction (CBED) measurements show that the crystal adopts the high‐symmetry *P6/mmm* structure (point group *D_6h_*). This apparent discrepancy of inversion symmetry breaking is resolved by considering intrinsic structural modifications arising from correlated disorder, as revealed by x‐ray diffuse‐scattering (DS) experiment by Korshunov et al. [29]. Their study demonstrates partial occupation of interlayer void sites by Yb atoms and Ge (Ge^T^) dimers, leading to stochastic packing along the *c* axis which produces anisotropic hexagonal diffuse rings, characteristic of a frustrated triangular lattice [29, 41, 42]. This packing arrangement gives rise to static, frustrated short‐range correlations extending over ∼30 Å in *ab*‐plane and ∼140 Å along *c* axis. Crucially, within these correlation lengths, Ge dimers tend to form small clusters in the *ab* plane and exhibit a preference for alternating arrangements with Yb along the *c* axis, making occupational disorder more coherent along the *c* axis. In addition, this occupational disorder induces correlated lattice relaxations, where neighbouring Ge^H^ atoms shift toward Ge dimers and away from Yb sites due to size mismatch [29]. This interplay of frustration and correlated displacement disorder enforces asymmetric atomic configurations over the correlation length scale. Although these structural modifications do not develop into long‐range order, they persist throughout the crystal and are sufficient to enable symmetry‐sensitive responses such as the NLHE. These local fluctuations remain undetected in our CBED experiment, which captures the average periodic structure within the nanoscale probe volume, but they can be directly resolved via diffuse scattering.

The pronounced anisotropy of the NLHE (Figure 3a–d), particularly the contrasting behavior between in‐plane (*I*∥*a*) and out‐of‐plane (*I*∥*c*) currents, directly reflects the anisotropic nature of correlated disorder as discussed above. The persistence of the out‐of‐plane current (*I*∥*c*) induced NLHE up to 350 K is consistent with the temperature‐independent nature of this correlated disorder‐driven inversion symmetry breaking, and is rooted in the geometrically frustrated configuration of the Yb/Ge sublattice. The greater coherence of Yb/Geᵀ dimer packing along the *c‐*axis, combined with local distortions from Ge^H^ relaxations, generates asymmetrical electronic structural modulation that can produce a sizeable BCD. Within these noncentrosymmetric regions, under an applied electric field, finite BCD generates an anomalous carrier velocity, producing a nonlinear transverse current [38]. Although the anomalous velocities from an ideal centrosymmetric bulk cancel exactly, the presence of spatially inhomogeneous inversion breaking associated with correlated disorder prevents complete cancellation on the nanoscale, giving rise to intrinsic NLHE. In addition, electrons traversing the interfaces between distorted clusters and the surrounding centrosymmetric matrix undergo disorder‐induced scattering, which also contributes to the nonlinear Hall response [37].

Since for pure intrinsic (BCD) induced NLHE *V_x_^2ω^* = 0, the finite *V_x_^2ω^* indicates significant disorder‐induced extrinsic scattering effects. Although this intrinsic disorder is static in nature, the amplitude of scattering is temperature dependent. In contrast, the more spatially random clustering of Geᵀ dimers within the *ab*‐plane can weaken the asymmetric Berry curvature distribution, consistent with the experimentally observed relation, *V_y_^2ω^* << *V_x_^2ω^* for in‐plane current (*I*∥*a*) induced transport. Beyond the correlation length, structural motifs fluctuate due to stochastic occupational disorder, creating additional asymmetric scattering centers. As confirmed by the scaling analysis, these extrinsic scattering processes dominate the amplitude of the NLHE signal, while the BCD contribution remains secondary and essentially temperature‐independent. Taken together, these observations indicate that the anisotropy of the frustrated triangular lattice favors BCD whose dominant component lies along the *c*‐axis, accounting for the much stronger out‐of‐plane nonlinear Hall response. Furthermore, the reduction of the NLHE signal at elevated temperatures, likely due to phonon‐assisted scattering, is supported by the anomalous broadening of out‐of‐plane phonon modes along the *Γ—A* direction observed in IXS measurements in Ref. [29]. This is consistent with the Debye temperature regime in our longitudinal resistivity analysis (Note S3).

**FIGURE 5:**
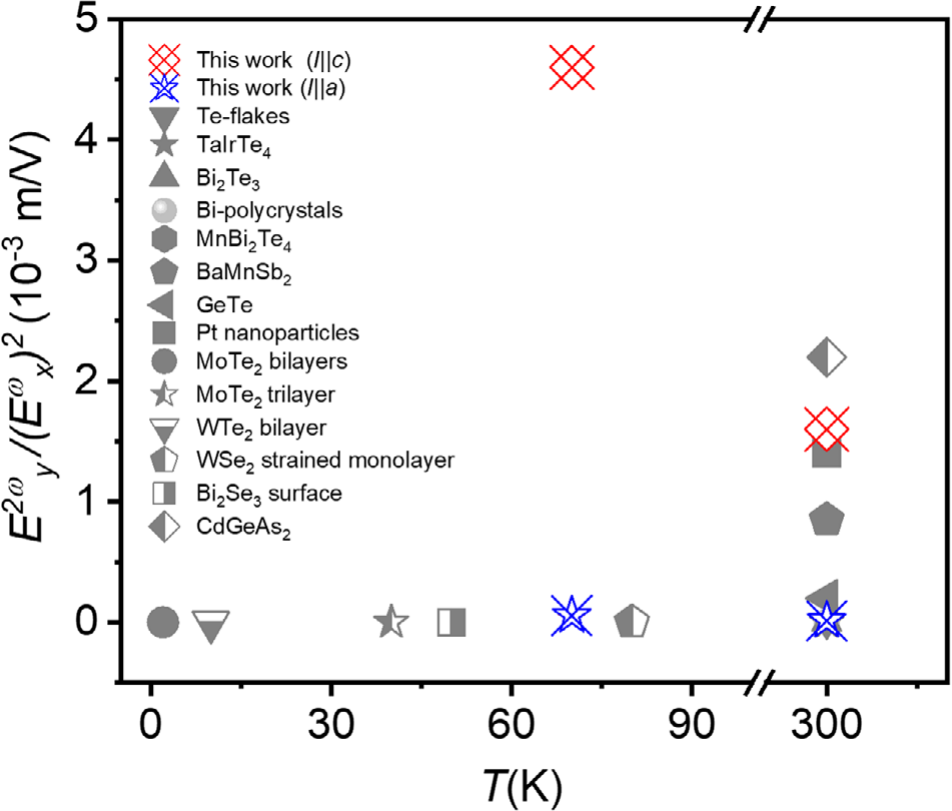
Comparison of the responsivity of various materials showed NLHE at different temperatures. References and responsivity values for the other materials shown here can be found in Table S4.

While prior studies have predominantly focused on the NLHE in 2D materials, practical applications have often been limited by low operating temperatures and modest NLHE responsivity. This responsivity, a key figure of merit in Hall systems, is typically below 10^−4^ m/V even in materials exhibiting room‐temperature NLHE [6, 22, 43, 44]. Achieving significant responsivity at ambient conditions in three‐dimensional systems has proven especially challenging, particularly for scattering‐induced NLHE. In contrast, for *I*||*c*, YbCo_6_Ge_6_ bulk single crystals achieves a record‐breaking responsivity of ∼4.6 × 10^−3^ m/V at 70 K (see Figure 5 and Table S4). This is the highest responsivity value documented for any nonlinear Hall material, surpassing the known record of ∼2.26 × 10^−3^ m/V for non‐centrosymmetric CdGeAs_2_ at room temperature. Moreover, this high‐performance state persists at room temperature, where we measure a robust ∼1.6 × 10^−3^ m/V responsivity value. This represents the largest room‐temperature response among all centrosymmetric nonlinear Hall systems and is competitive with the best non‐centrosymmetric CdGeAs_2_ [45]. Critically, this NLHE persists well above room temperature with strong anisotropy, yielding a large nonlinear Hall angle and high responsivity that make YbCo_6_Ge_6_ a promising platform for rectifiers, frequency multipliers, and nonlinear optoelectronic devices requiring stable, cryogen‐free operation. The pronounced anisotropy between out‐of‐plane and in‐plane responses further enables directional control of the nonlinear signal, a valuable feature for ambient‐temperature applications. Moreover, the ability to harness the material's structural attributes, where short‐range correlated motifs underpin the observed transport properties, establishes a new materials design pathway for nonlinear electronics, showing that intrinsic structural correlations can serve as a functional asset rather than a limitation in three‐dimensional quantum materials.

## 4 Conclusions

The nonlinear Hall effect (NLHE) in YbCo_6_Ge_6_ arises from distortions in the kagome lattice that couple strongly to the anisotropic electronic structure, resulting in a pronounced, direction‐dependent response dominated by out‐of‐plane (*I*∥*c*) transport. The nonlinear signal is primarily governed by scattering‐driven mechanisms: static disorder dominates at low temperatures, while phonon‐assisted processes become significant at higher temperatures. Notably, the NLHE remains robust at temperatures above room temperature. These results demonstrate a direct relationship between correlated structural distortions and nonlinear transport in kagome systems, underscoring the potential to engineer electronic responses through lattice manipulation. The large, anisotropic, and thermally stable nonlinear Hall response identifies YbCo_6_Ge_6_ as a promising platform for energy‐efficient rectification, high‐frequency detection, and directionally encoded electronic functionalities, as well as for the development of strain and disorder‐tunable device architectures.

## 5 Methods

### 5.1 Crystal Growth

High‐quality single crystals of YbCo_6_Ge_6_were grown using a Sn‐flux method. Stoichiometric amounts of Yb, Co, and Ge, along with Sn flux (molar ratio Yb:Co:Ge:Sn = 3:2:7:52), were sealed in an evacuated quartz tube. The ampoule was heated to 1373 K in 12 h and held for 24 h to ensure complete homogenization, followed by slow cooling to 1100 K at a rate of 2 K/h. Excess Sn flux was removed by centrifugation, yielding needle‐shaped single crystals with typical dimensions of 2–3 mm in length.

### 5.2 Device Fabrication

Crystals were oriented and loaded in a focused ion beam (FIB) system (Thermo Scientific Helios 5 dual‐beam SEM/FIB). Hall‐bar devices with micron‐scale dimensions were patterned using a Ga focused ion beam (30 kV) at a low fluence of <1 × 10^14^ ions/cm^2^, corresponding to a penetration depth of ∼20 nm. Lamellae were polished and lifted out from single‐crystal specimens along the desired crystallographic directions. The ion‐beam‐damaged layer is confined to the near‐surface region; compared with the bulk crystal. Its contribution to conductivity is insignificant. The devices were then contacted to the pre‐patterned SiO_2_/Si substrate using the Pt deposition function. The pre‐patterned electrodes are 180 nm Pd plus 20 nm Ti. The contacts between the sample and pre‐pattern are ∼1 um Pt. The measurement geometries of the FIB‐patterned devices are: the alternating current flowing along the *a* axis, and the magnetic field applying along the *c* axis; or current flowing along the *c* axis and field applying along the *a* axis.

### 5.3 Transport Measurements

Electrical transport properties were measured using a standard lock‐in technique (Stanford Research Systems SR830) with an excitation frequency of 37 Hz. The sample was mounted in a Physical Property Measurement System (PPMS, Quantum Design) equipped with a 7 T superconducting magnet, allowing precise control of temperature (2–300 K) and magnetic field (0‐7 T) during measurements. Four probe configurations were employed to ensure accurate resistivity measurements while minimizing contact resistance effects. All the measurements were done at frequency of 37 Hz. Magnetometry measurements were conducted using a vibration sample magnetometer (VSM) mounted on a 12 T PPMS Dynacool. During measurements, sample was mounted on a quartz sample holder using Kapton tape. Zero‐field cooling mode was employed: (1) sample was cooling to base temperature; (2) the measurement field (500 Oe) was applied via linear mode; (3) magnetization data were collecting during heating to 300 K.

### 5.4 Structural Characterizations (TEM)

Specimen for transmission electron microscopy (TEM) was prepared using a standard lift‐out technique in a Thermo Scientific Helios 5 UX FIB‐SEM, with a carbon coating applied to protect the native surface. Subsequent STEM analysis was performed on a dual aberration‐corrected FEI Titan [3] 80–300 at 300 kV with a convergence angle of 21 mrad. Using a camera length of 91 mm, HAADF, ABF, and BF images were acquired simultaneously to provide complementary contrast from atomic columns, light elements, and crystal defects. For diffraction analysis, zero‐loss energy‐filtered CBED patterns were recorded on a CCD with a Gatan Tridiem 863 P image filter (10 eV slit width), and 4D‐STEM datasets were acquired using an FEI EMPAD G3 detector with a convergence angle of 3 mrad to prevent disc overlap. Finally, EDX spectroscopy was conducted on the Titan using an Oxford Instruments Ultimax detector with the specimen tilted approximately 15° to enhance x‐ray yields.

### 5.5 Computational Methods

To examine the electronic band structure of YbCo_6_Ge_6_, first‐principles calculations based on density functional theory (DFT) were performed using the Vienna Ab initio Simulation Package (VASP) [32, 46, 47]. Band structure and phonon dispersion were calculated for the pristine, centrosymmetric hexagonal *P6/mmm* phase of YbCo_6_Ge_6_, without disorder or partial occupancy. The exchange‐correlation effects were described within the generalized gradient approximation (GGA) using the Perdew‐Burke‐Ernzerhof functional (PBE) formulation [47, 48, 49, 50], with an energy cut‐off of 350 eV to ensure computational accuracy. Spin‐orbit coupling (SOC) was explicitly included in the calculations to account for relativistic effects. Structural optimization of YbCo_6_Ge_6_ was conducted until the Hellmann‐Feynman forces acting on each atom were reduced below 0.01 eV/Å, with the total energy convergence threshold set to 10^−6^ eV. The Brillouin zone was performed using a Γ‐centered 10×10×6 k‐point mesh. The phonon dispersions were computed using Phonopy interfaced with VASP, using 3 × 3 × 2 supercells. An energy cutoff of 350 eV and a k‐point mesh of 4 × 4 × 3 were used.

## Author Contributions

**Weiyao Zhao**: investigation, writing – review and editing, methodology, conceptualization. **Junlin Yan**: investigation, writing – review and editing. **Thanh Tung Huynh**: investigation, writing – review and editing, methodology. **Yong Fang**: investigation, writing – review and editing, funding acquisition, supervision. **Qurat Ul Ain**: writing – review and editing, writing – original draft, investigation, conceptualization, formal analysis, methodology. **Yuefeng Yin**: investigation, methodology, writing – review and editing. **Fang Tang**: investigation, writing – review and editing. **Timothy Petersen**: investigation, writing – review and editing. **Yang Liu**: investigation, writing – review and editing. **Jacek Jasieniak**: funding acquisition, writing – review and editing, supervision, project administration. **Nikhil V. Medhekar**: conceptualization, writing – review and editing, investigation, funding acquisition. **Julie Karel**: conceptualization, investigation, funding acquisition, writing – original draft, writing – review and editing, methodology, supervision, resources.

## Conflicts of Interest

The authors declare no conflicts of interest.

## Supporting information




**Supporting File**: advs77434‐sup‐0001‐SuppMat.docx.

## Data Availability

The data that support the findings of this study are available from the corresponding author upon reasonable request.
