# Supplementary material for: Disorder Driven Anisotropic Nonlinear Hall Effect in the Kagome Metal YbCo6Ge6 at Room Temperature
Source: Adv Sci (Weinh). 2026 Aug 27;13(63):e77434. doi: 10.1002/advs.77434 (PMC13521198; doi:10.1002/advs.77434)
Supplement: Supplementary file 1 [file ADVS-13-e77434-s001.docx]

***Supplementary Information for***

**Disorder driven Anisotropic Nonlinear Hall Effect in kagome metal YbCo_6_Ge_6_ at Room Temperature**

Qurat Ul Ain^1^, Weiyao Zhao^1^, Thanh Tung Huynh^1^, Yuefeng Yin^1^, Fang Tang^2^, Yong Fang^2^, Junlin Yan^1^, Jacek Jasieniak^1^, Timothy Petersen^3^, Yang Liu^3^, Nikhil V Medhekar^1^, Julie Karel^1*^

^1^ Department of Materials Science & Engineering, Monash University, Clayton VIC 3800, Australia

^2^ Jiangsu Laboratory of Advanced Functional Materials, School of Electronic and Information Engineering, Suzhou University of Technology, Changshu 215500, China

^3^ Monash Centre for Electron Microscopy (MCEM), Monash University, Clayton VIC 3800, Australia

^4^ School of Physics and Astronomy, Monash University Clayton VIC 3800, Australia

**Keywords:** nonlinear Hall effect, Berry curvature, skew scattering, kagome metal

* Corresponding author Email address: [julie.karel@monash.edu](mailto:julie.karel@monash.edu)

1. ***Crystal homogeneity and defect distribution***

Energy Dispersive X-ray Spectroscopy (EDX) confirmed that the YbCo_6_Ge_6_ single crystals are nearly stoichiometric. Transmission electron microscopy (TEM) analysis indicates that the crystal matrix is predominantly defect-free; however, examination at lower magnification identified a sparse distribution of minor defects across the lamella prepared by focused ion beam (FIB) milling. Almost no defects were detected when imaging along the [0001] zone axis. As presented in Supplemental Figure 1(a, b), a pair of potentially interacting defects is highlighted by annular bright-field (ABF) contrast. The absence of mass-thickness contrast in the corresponding high-angle annular dark-field (HAADF) image confirms that these features are structural, thereby excluding the presence of chemical precipitates. Atomic resolution imaging of these defects (Supplemental Figure 1(c, d)) reveals their crystallographic nature. Further analysis of the strain and in-plane rotation around the defect (Supplemental Figure 1(e, f)) demonstrates local rotation distortions in the lattice, indicative of a strain field associated with a screw dislocation. The corresponding power spectra are provided in Supplemental Figure 1(g, h). When the crystal was oriented along an orthogonal direction from the <11-20> family, an array of extended structural defects was observed near the centre of the lamella (Supplemental Figure 1(i, j)), representing the only other significant defects across the specimen. Atomic resolution close-up views of these nearly horizontal defects (Supplemental Figure 1(k, l)) yielded limited structural information. Roaming local diffractograms from small regions of interest showed no evidence of significant strain.

**Supplemental Figure 1:** Supplemental Figure 1: Scanning transmission electron microscopy analysis of YbCo₆Ge₆ single crystals. (a) Annular bright-field (ABF) and (b) high-angle annular dark-field (HAADF) images of the crystal oriented along the [0001] zone axis. The absence of mass-thickness contrast in the HAADF image confirms that the few observed defects are structural in nature, rather than chemical precipitates. (c) Higher magnification ABF image of the defect pair shown in (a), with (d) a corresponding HAADF close-up of the leftmost defect, further supporting the structural origin of the feature. (e, f) Analysis of the defect in (d) reveals local in-plane rotation of nearly 4 degrees, possibly identifying an isolated screw dislocation. (g, h) Power spectra corresponding to the regions in (e) and (f), respectively. (i) Bright-field (BF) and (j) simultaneous HAADF images of the crystal oriented along a <11-20> zone axis. A sparse array of extended defects was observed near the center of the lamella, representing the only other noticeable imperfections. (k, l) ABF and HAADF close-up views of one of the horizontal defects from (i, j). These atomic-resolution images, along with diffractograms from small regions of interest (not shown), did not reveal substantial local strain, suggesting these are relatively low-energy structural defects. TEM lamella were prepared from the same crystal piece from which sample S9 and S10 were prepared for transport measurements.

2. ***SQUID Magnetometry Analysis***

Magnetic susceptibility measurements of YbCo_6_Ge_6_ as a function of temperature are presented in Supplemental Fig 2 for an applied field of 0.05T along *H*||*c* and *H*||*a*. The absence of any sharp anomaly or transition down to 2 K confirms the lack of long-range order and supports the paramagnetic nature of YbCo_6_Ge_6_ single crystals. Primarily, the magnetic response in this compound is expected to come from Yb^3+^, while Co is nonmagnetic in this system. The high temperature data were fitted with the modified Curie-Weiss law (inset of supplemental Fig. 2):

$$\chi=\frac{{\mu_{0}N}_{A}\mu_{eff}^{2}}{3k_{B}(T-\Theta_{CW})}+\chi_{0}$$

where $\chi_{0}$ is the temperature independent susceptibility, $\Theta_{CW}$ is the Curie-Weiss temperature and *μ_eff_* is the effective magnetic moment. Fitting the *χ^-1^*(*T*) yield *μ_eff_* of 3.1(8) μ_B_ and 3.6(1) μ_B_ for *H*||*c* and *H*||*a* respectively, both the values are smaller than the expected free-ion value of 4.54 μ_B_ for Yb^3+^. The extracted $\Theta_{CW}$ are -9.6(7) K and -43.2(12) K indicating antiferromagnetic correlation and notable magnetic anisotropy, $\chi_{0}$ has minor contributions of 7.3×10^-4^ emu/ mol and 1.79×10^-3^ emu/mol for *H*||*c* and *H*||*a* respectively. Despite the absence of long-range magnetic order down to 2 K, the reduced effective magnetic moment *μ_eff_*, combined with the negative $\Theta_{CW}$, suggests the presence of underlying magnetic correlations and anisotropic exchange interactions in YbCo_6_Ge_6_. This behavior points to a magnetically correlated paramagnetic state, likely influenced by crystal electric field (CEF) effects and hybridization between localized 4f states and conduction electrons, which modify the magnetic response from that of a free Yb^3+^ ion ^1-3^.

**Supplemental Figure 2.** Temperature dependence of magnetic susceptibility *χ*(*T*) of YbCo_6_Ge_6_ single crystals at an applied magnetic field of μ_0_*H* = 0.05 T along the two crystallographic axes *a-*axis and *c-*axis. Inset: The inset shows the inverse susceptibility *χ^-1^*(*T*) and the red solid lines are the Curie-Weiss fits in high temperature region.

All the transport measurements shown in the main text and corresponding SI are for sample S9 and S10 for *I||c* and *I||a* respectively.

3. ***Bloch-Grüneisen (BG) model Analysis of longitudinal resistivity***

The red solid lines in the main text of Fig. 1(d) are the fits to Bloch-Grüneisen (BG) model over the complete temperature region:

$\rho(T,0) = \rho_{0} + \rho_{1}T^{2} +4C\Theta_{D} ({\frac{T}{\Theta_{D}})}^{5}\int_{0}^{\Theta_{D}/T} \frac{x^{5}e^{x}}{{(e}^{x}-1)(1-e^{-x})}dx$	(1)

Supplemental Table 1: Parameters derived from the Bloch-Grüneisen (BG) model fits of longitudinal resistivity.

| *Direction* | $\boldsymbol{\rho}_{\boldsymbol{0}}$ (μΩ cm) | $\boldsymbol{\rho}_{\boldsymbol{1}}$ (μΩ cm K^-2^) | *C (*μΩ cm K^−5^) | $\boldsymbol{\Theta}_{\mathbf{D}}$(K) | RRR (=$\boldsymbol{\rho}_{\boldsymbol{300}\mathbf{K}}\boldsymbol{/}\boldsymbol{\rho}_{\boldsymbol{2}\mathbf{K}}$) |
| --- | --- | --- | --- | --- | --- |
| *I\|\|c* | 21.83(1) | -1.09×10^-4^ (3) | 0.16 (2) | 222.1(1) | 2.1 |
| *I\|\|a* | 4.41(2) | -3.38×10^-6^ (5) | 0.02 (3) | 193.8 (4) | 2.7 |

The first term on the right is a residual resistivity, 𝜌_0_, attributed to impurity scattering. The second term, $\rho_{1}$ (in μΩ cm K^−2^), is attributed to electron-electron scattering (Fermi liquid term), and the third term describes the electron-phonon scattering, $\Theta_{D}$ is the Debye temperature, and *C* is a constant. The deduced parameters are summarized in Table 1. The negative Fermi liquid coefficients ($\rho_{1}$) suggest deviations from conventional Fermi liquid behavior. Such a sign inversion may reflect interband scattering, band hybridization, or the presence of topologically nontrivial bands near *E_F_*, which are commonly observed in correlated and semimetallic systems ^4-6^. The small residual resistivity ratio (RRR = $\rho_{300K}/\rho_{2K}$) hints at a disorder effect but preserves metallicity.

4. ***Hall Resistivity***

The *ρ_xy_* linearly depends on *H* for the field applied along *H*||*c* (*I*||*a*) and along *H*||*a* (*I*||*c*). To deduce the Hall coefficient (R_H_), the raw data was fitted with a linear equation, SI Fig 4 (a, b) represents the linear fits the raw data is shown in SI Fig. 3 (the blue open circles are the experimental data points, and the red solid lines are the straight line fits at 10 K). The negative slopes in SI Fig. 4 (a,b) indicate the electrons dominated conduction. The carrier densities (*n =* 1/*e*R_H_, where *e* is elemental charge) and mobilities (*μ* = R_H_/*ρ_xx_*) are presented in SI Fig. 4(c). The extracted carrier mobilities show anisotropy, with higher mobility observed for out of plane current flow (*I*∥*c*) compared to in-plane (*I*∥*a*), consistent with the material's electronic structure. This anisotropy directly correlates with the resistivity measurements, where the lower in-plane mobility corresponds to the higher *ρₓₓ* observed for *I*∥*a*, attributed to charge carrier localization in the Kagome-derived flat band and HOSP near the Fermi level. In contrast, the steeper band dispersion along the *c*-axis facilitates higher mobility for *I*∥*c* transport.

**Supplemental Figure 3:** Hall resistivity raw data with applied field along two orientations (a) *H*||*a* (sample, S9) and (b) *H*||*c* (sample, S10). The blue circles are the experimental data points, and the red solid lines are linear fits.

**Supplementary Figure 4.** (a, b) Hall resistivity (*ρ_xy_*) along *H*||*c* (*I*||*a*) (sample, S10) and *H*||*a* (*I*||*c*) (sample, S9). (c) Carrier concentration and mobilities for *H*||*c* (*I*||*a*) and *H*||*a* (*I*||*c*)

5. ***Exclusion of external effects irrelevant to contribution to NLHE***

The secondary electron (SE) image of the fabricated device as shown in the inset of Fig. 2(a) of main text confirms its structural integrity. The image reveals uniform channel dimensions and well-aligned contact electrodes (Pt), with clean edges and sharp interfaces, which are essential for minimizing extrinsic scattering effects in electronic transport studies. Several external effects such as asymmetric sample shape, thermoelectric effect and contact junction effect. Multiple devices yielded consistent results, and the stark anisotropy between in plane and out of plane responses excludes contact effects as the origin. AC measurements at the second harmonic frequency effectively cancel thermoelectric contributions. The smooth evolution of nonlinear signals from 2 - 300 K and their well-behaved current dependence rule out Joule heating effects. Also, the temperature gradient induced by joule heating would be largest in the longitudinal direction (*V_x_^2ω^*), which is not the case for YbCo_6_Ge_6_.

6. ***Temperature dependence of NLHE***

Supplemental Fig. 5 illustrates the temperature dependence of NLHE, exhibiting a non-monotonous increase in *V_y_^2ω^* when current flows out of plane *I*∥*c*, followed by a broad hump above 100 K and a maximum value around 200 K. Further increases in temperature lead to a decrease in *V_y_^2ω^*, possibly due to full phonon activation modes above this temperature. Interestingly, the Berry curvature dipole strength given as $\frac{E_{y}^{2\omega}}{{{(E}_{x}^{\omega})}^{2}}$ showed a peak at ~70 K, indicating the BCD is maximum at this temperature (see Fig. 2c main text). The NL longitudinal response *V_x_^2ω^* at this temperature vanishes, as can be seen in Supplemental Fig. 5(a), black panel, indicating that the total contribution at this temperature is purely intrinsic. However, on the other side, for current flowing out of plane *I*∥*a*, where *V_x_^2ω^* is always greater than *V_y_^2ω^*, showing the primary NL response is coming from extrinsic scattering effects. *V_y_^2ω^* appears to be saturated at all temperatures above 100 K (See Supplemental Fig. 5(b)).

**Supplemental Figure 5.** Temperature evolution of *V_y_^2ω^* and *V_x_^2ω^* for (a) *I*∥*c* (sample, S9) and (b) *I*∥*a* (sample, S10) at 2.5mA. The solid lines are guide to eyes.

7. ***Magnetic Field dependence of NLHE***

NLHE was measured as a function of applied magnetic field (μ_0_H) in the range of -6 T to 6 T. Over the whole range, the NLHE measured for current flowing along the two orientations was found to be independent of μ_0_*H* (see Supplemental Figure 6). In YbCo_6_Ge_6_, time-reversal (TR) symmetry is preserved, and even with the externally applied magnetic field the NL response remains independent. Therefore the intrinsic contribution to the NLHE in this case can arise from Berry curvature dipole (BCD) and not quantum metric dipole (QMD).

**Supplemental Figure 6:** NLHE observed as a function of μ_0_*H* for two orientations *I*∥*c* (sample, S9) and *I*∥*a* (sample, S10) at 2.5mA. The data shown in the two panels were acquired at different temperatures solely due to experimental convenience during a single measurement session, without any deliberate physical motivation.

8. ***Nonlinear Hall Conductivity***

A peak is observed in the nonlinear Hall conductivity $\sigma_{y}^{2\omega}= \frac{{\sigma_{xx}V}_{y}^{2\omega}L^{2}}{{{(V}_{x}^{\omega})}^{2}W}$ (see SI Fig. 7(d)) but at slightly lower temperature compared to $\frac{E_{y}^{2\omega}}{{{(E}_{x}^{\omega})}^{2}}$. This discrepancy is likely due to the $\sigma_{y}^{2\omega}$ dependence on $\sigma_{xx}$ along with **D,** since $\sigma_{y}^{2\omega}$ ∝ **D**$\sigma_{xx}$ ^7^. We have shown temperature evolution of $V_{x}^{\omega}$, $\sigma_{xx}$, and $V_{y}^{2\omega}$ along with $\sigma_{y}^{2\omega}$ to better highlight this point.

**Supplemental Figure 7:** (a) Temperature dependence of first harmonic $V_{x}^{\omega}$. (b) Temperature dependence of longitudinal conductivity $\sigma_{xx}$. (c) Temperature evolution of second harmonic *V_y_^2ω^* (d) Experimental nonlinear Hall conductivity for current flowing in plane *I*∥*c* and out of plane *I*∥*a*. The solid lines are the nonlinear Hall conductivity calculated from sum of *α* contributions. Data shown for *I*∥*c* is for sample S9 and for *I*∥*a* from sample S10.

9 ***Scaling Analysis***

The scaling analysis was conducted by plotting $\frac{E_{y}^{2\omega}}{{{(E}_{x}^{\omega})}^{2}}$vs. normalized longitudinal conductivity $\frac{\sigma_{xx}}{\sigma_{0}}$, where $\sigma_{0}=$ *σ* (2 K) (see Fig. 4(a, b)). According to theory, $\frac{E_{y}^{2\omega}}{{{(E}_{x}^{\omega})}^{2}}$ can be scaled as:

$\frac{E_{y}^{2\omega}}{{{(E}_{x}^{\omega})}^{2}}=\frac{\sigma_{y}^{2\omega}}{\sigma_{xx}}= \alpha_{0}+\alpha_{1}(\frac{\sigma_{xx}}{\sigma_{0}})+{\alpha_{2}(\frac{\sigma_{xx}}{\sigma_{0}})}^{2}$	(1)

where $\alpha_{i}$ (*i* = 0, 1, 2) indicate the mixed contributions from intrinsic, extrinsic side jump, and extrinsic skew scattering which is further categorised into Gaussian and non-Gaussian skew scattering mechanisms. Fitting parameters in Eq. 1 are defined as; $\alpha_{0}= C^{INT}+C_{1}^{SJ}+C_{11}^{SK,1}$; $\alpha_{1}= C_{01}^{SK,1}-2C_{11}^{SK,1}+C_{0}^{SJ}-C_{1}^{SJ}$ and $\alpha_{2}=C^{SK,2} \sigma_{0}+C_{00}^{SK,1}+C_{11}^{SK,1}-C_{01}^{SK,1}$, where $C^{INT}$ indicate the intrinsic mechanism, *SJ* denotes side jump and *SK* is used for skew scattering, *SK,*1 and *SK,* 2 indicate the Gaussian and non-Gaussian skew scattering, the subscript 0 and 1 indicate the static (impurity, defects) and dynamic (phonon) scatterings respectively. Simplifying the above, the mechanisms involved in $\alpha_{0}$ are intrinsic (e.g., BCD/QMD), dynamic electron-phonon scatterings (*SJ*, *SK*,1); $\alpha_{1}$ are mixed static and dynamic scatterings (*SK*,1 and *SJ*); and $\alpha_{2}$ are mixed Gaussian and non-Gaussian skew scatterings (*SK*,2 and *SK*,1). Fitting parameters derived are summarized in supplemental table 2. This scaling analysis follows the same functional form as previously established for other nonlinear Hall systems, including Bi thin films ^8^, twisted bilayer graphene ^9^, Bi_2_Te_3_ ^10^. Remarkably, despite the differences in material composition, dimensionality, and band structure, the same α-parameter scaling emerges across all these systems, suggesting that this empirical relation captures a universal feature of the nonlinear Hall response.

Supplemental Table 2: Parameters derived from the scaling analysis of NLHE using *V_y_^2ω^* vs. *T* data. Data shown for *I*∥*c* is from sample S9 and for *I*∥*a* is from sample S10.

| *Direction* | $\boldsymbol{\alpha}_{\boldsymbol{0}}$ (m/V) | $\boldsymbol{\alpha}_{\boldsymbol{1}}$ (m/V) | $\boldsymbol{\alpha}_{\boldsymbol{2}}$ *(*m/V) |
| --- | --- | --- | --- |
| *I\|\|c* | -1.224×10^-2^ ± 1.322×10^-4^ | 4.102×10^-2^ ± 3.753×10^-4^ | -2.537×10^-2^ ± 2.542×10^-4^ |
| *I\|\|a* | -1.027×10^-4^ ± 1.948×10^-6^ | 4.151×10^-4^ ± 6.353×10^-6^ | -2.829×10^-4^ ± 4.732×10^-6^ |

To further validate the scaling behavior of the nonlinear Hall effect (NLHE), we performed an additional analysis using the current-voltage (*IV*) curves measured at various temperatures for the *I*||*c* configuration (main text Figure 3(c)). For each temperature, we extracted the highest current value and calculated the ratio $\frac{E_{y}^{2\omega}}{{{(E}_{x}^{\omega})}^{2}}$. As shown in Supplemental Figure 8(a), this analysis reveals a clear quadratic dependence as described in Eq(1). The scaling *α*-parameters tabulated in SI table 3 and the nonlinear conductivity coefficient $\sigma_{y}^{2\omega}$ (SI Figure 8(b)) extracted from this analysis are in excellent agreement with those obtained from the temperature-dependent $V_{y}^{2\omega}$data (Supplemental Table 2), confirming the robustness of our scaling approach.

Supplemental Table 3: Summary of fitting parameters extracted from the scaling analysis of the nonlinear Hall effect (NLHE) using the *IV-*curves acquired at various temperatures (main text Figure 3(b)) for sample S9.

| *Direction* | $\boldsymbol{\alpha}_{\boldsymbol{0}}$ (m/V) | $\boldsymbol{\alpha}_{\boldsymbol{1}}$ (m/V) | $\boldsymbol{\alpha}_{\boldsymbol{2}}$ *(*m/V) |
| --- | --- | --- | --- |
| *I\|\|c* | -1.211×10^-2^ ± 1.700×10^-3^ | 3.984×10^-2^ ± 4.900×10^-3^ | -2.359×10^-2^ ± 3.280×10^-3^ |

**Supplemental Figure 8**. Scaling analysis for YbCo_6_Ge_6_ for *I*||*c* using *IV*-curve data for maximum I values (a) Parabolic fitting of $\frac{E_{y}^{2\omega}}{{{(E}_{x}^{\omega})}^{2}}$vs. $\frac{\sigma_{xx}}{\sigma_{0}}$ for *I*∥*c.* (b) Weights of terms in Eq. (3) calculated for *I*∥*c* contributing to $\sigma_{y}^{2\omega}$. Data shown for *I*∥*c* is for sample S9.

**Supplemental Figure 9:** Scaling analysis for YbCo_6_Ge_6_. (a, b) Parabolic fitting (red solid line) of $\frac{E_{y}^{2\omega}}{{{(E}_{x}^{\omega})}^{2}}$vs. $\frac{\sigma_{xx}}{\sigma_{0}}$ (black open circles) for *I*∥*c* (a) and *I*∥*a* (b). Weights of terms in SI Eq. (1) calculated for *I*∥*c* (a) and *I*∥*a* (b) contributing to $\frac{E_{y}^{2\omega}}{{{(E}_{x}^{\omega})}^{2}}$at specific temperatures.

10. ***Band structure***

The calculated electronic band structure for the pristine structure *P*6/*mmm* is shown in SI Fig. 10(b). It features groups of flatbands (FB) primarily derived from Co *d* orbitals, alongside two prominent higher-order saddle points (HOSPs), labeled ξ₁ and ξ₂, located near the *L* point at approximately 0.058 eV and 0.185 eV, respectively. These features are consistent with those reported in Ref. ^11^, though observed here at slightly higher energies. Additionally, the ξ₁ saddle point gives rise to a nearly flatband (FB) along the *L-H* direction. Dirac points (DPs) present at the *H* point are found to be gapped due to the effects of spin-orbit coupling (SOC). The phonon band structure (SI Fig. 10(c)) indicate the presence of imaginary modes resulting in low symmetry phases induced by lattice distortions similar to Ref. ^11^ which can induce nonlinear transport in the material.

**Supplemental Figure 10:** Band structure for YbCo_6_Ge_6_ in *P6mmm* phase. (a) Schematic illustration of the Brillouin zone with high-symmetry points labelled. (b) Calculated electronic band structure of pristine *P6/mmm* YbCo_6_Ge_6_ including spin-orbit coupling (SOC), showing flatbands (FB), higher-order saddle points (HOSP) ξ₁, and ξ₂ near *L*, and SOC-gapped Dirac points at *H*. (c) Phonon band structure with imaginary frequency depicted as negative values.

Band structure and phonon dispersion were calculated for the pristine, centrosymmetric hexagonal P6/mmm phase of YbCo6Ge6, without disorder or partial occupancy. The phonon dispersions were computed using Phonopy interfaced with VASP ^12, 13^, using 3×3×2 supercells. An energy cutoff of 350 eV and a k-point mesh of 4×4×3 were used.

**Supplemental Figure 11.** Results of convergence tests for (a) energy cutoff and (b) k-point mesh. Red circles indicate the values chosen for the DFT calculations.

SI Table 4: Responsivity of various materials.

| *Material* | *Dimensionality* (D) | *Temperature* (K) | *2^nd^ order voltage response* | *Responsivity*  (m/V) | Ref. |
| --- | --- | --- | --- | --- | --- |
| YbCo_6_Ge_6_ | 3 (*I*\|\|*c*) | 70 | ~8.4μV at 2.5mA | ~4.6×10^-3^ | This work (sample S9) |
| YbCo_6_Ge_6_ | 3 (*I*\|\|*c*) | 300 | ~9.6μV at 2.5mA | ~1.6×10^-3^ | This work (sample S9) |
| YbCo_6_Ge_6_ | 3 (*I*\|\|*a*) | 70 | ~2.1μV at 2.5mA | ~5.5×10^-5^ | This work (sample S10) |
| YbCo_6_Ge_6_ | 3 (*I*\|\|*c*) | 300 | ~2.7μV at 2.5mA | ~1.4×10^-5^ | This work (sample S10) |
| MoTe_2_ bilayer | 2 | 2 | 125μV at 97μA | 7×10^-9^ | ^14^ |
| MoTe_2_ trilayer | 2 | 40 | 6.5μV at 93μA | 1.2×10^-8^ | ^14^ |
| Few layer WTe_2_ | 2 | 10 | 30μV at 600μA | 1.4×10^-7^ | ^15^ |
| WSe_2_ Strained monolayer | 2 | 80 | 20μV at 4.5μA | 6.2×10^-7^ | ^16^ |
| Bi_2_Se_3_ | 2 | 50 | 2μV at 1500μA | 0.8×10^-9^ | ^17^ |
| TaIrTe_4_ | 3 | 300 | 120μV at 600μA | 2.5×10^-8^ | ^18^ |
| MnBi_2_Te_4_ | 2 | 300 | 225μV at 250μA | 3×10^-10^ | ^19^ |
| Bi-polycrystalline films | 2 | 300 | 3μV at 60μA | 2.4×10^-9^ | ^8^ |
| BaMnSb_2_ | 3 | 300 | 250μV at 100μA | 8.5×10^-4^ | ^7^ |
| Te - flakes | 2 | 300 | 2800μV at 50μA | 8×10^-8^ | ^20^ |
| GeTe | 3 | 300 | 1μV at 5mA | 2×10^-4^ | ^21^ |
| Pt nanoparticles | 3 | 300 | 90μV at 95μA | 1.4×10^-4^ | ^22^ |
| CdGeAs_4_ | 3 | 300 | 1200μV at10mA | 2.2×10^-3^ | ^23^ |
| Bi_2_Te_3_ | 2 | 300 | 250μV at 900μA | 3.5×10^-9^ | ^24^ |

11. ***Reproducibility***

To check the reproducibility of the observed NLHE, transport measurements were performed on additional devices, and similar results were obtained.

**Supplemental Figure 12:** (a)Temperature dependent longitudinal resistivity (*ρ_xx_*) measured with the applied current parallel to the *c*-axis (black circles for device S6) and *a*-axis (blue circles for device S5). (b-d) Nonlinear electronic transport in device S5 and S6. (b) Nonlinear Hall voltage *V_y_^2ω^* as a function of driving current *I^ω^* at 2 K for *I*||*c* solid blue sphere and solid black sphere for *I*||*a*. (c) Temperature dependence of nonlinear Hall voltage *V_y_^2ω^* for *I*||*c* and *I*||*a*. The solid line guide to eye obtained by the average of raw data points. (d) Magnetic field dependence of NLHE *V_y_^2ω^* at 2 K for *I*||*c* and *I*||*a*.

**Supplemental Figure 13:** Scaling analysis for device S6 for *I*||*c*. (a) A few interpolated nonlinear Hall voltage *V_y_^2ω^* points as a function of temperature from the solid black line in SI Fig. 12(c). (b) Temperature dependence of NLHE strength $\frac{E_{y}^{2\omega}}{{{(E}_{x}^{\omega})}^{2}}$ known as responsivity for *I*||*c* (S6) calculated in a similar way as mentioned in main text. (c) Parabolic fitting of SI Eq. (1) $\frac{E_{y}^{2\omega}}{{{(E}_{x}^{\omega})}^{2}}$vs. $\frac{\sigma_{xx}}{\sigma_{0}}$ for *I*∥*c* (S6). (d) Weights of terms in Eq. (3) calculated for *I*∥*c* contributing to $\sigma_{y}^{2\omega}$. The fitting parameters obtained were $\alpha_{0}=-0.072 \pm0.009\left( \frac{m}{V} \right), \alpha_{1}=0.225 \pm0.026\left( \frac{m}{V} \right) and \alpha_{2}=0.102 \pm0.018\left( \frac{m}{V} \right)$, presence of three components indicate contributions from intrinsic and extrinsic scattering similar to sample S9 presented in details in main text. The nonlinear Hall conductivity $\sigma_{y}^{2\omega}$ reveals the dominant contribution is scattering however, the nonzero $\alpha_{0}$ term reflects the presence of intrinsic BCD contributions.

**Supplemental Figure 14:** (a)Temperature dependent longitudinal resistivity (*ρ_xx_*) measured with the applied current parallel to the *c*-axis for device S4. (b-d) Nonlinear electronic transport in device S4. (b) Temperature dependence of nonlinear Hall voltage *V_y_^2ω^* for *I*||*c* (S4). (c) Temperature dependence of NLHE strength $\frac{E_{y}^{2\omega}}{{{(E}_{x}^{\omega})}^{2}}$ known as responsivity for *I*||*c* (S4). (d) Parabolic fitting of SI Eq. (1) $\frac{E_{y}^{2\omega}}{{{(E}_{x}^{\omega})}^{2}}$vs. $\frac{\sigma_{xx}}{\sigma_{0}}$ for *I*∥*c* (S4). (e) Weights of terms in Eq. (3) calculated for *I*∥*c* contributing to $\sigma_{y}^{2\omega}$. The fitting parameters obtained were $\alpha_{0}$ = 1.02×10^-2^ ± 6.74×10^-4^(m/V), $\alpha_{1}$= 3.54×10^-2^ ± 2.02 × 10^-3^(m/V), and $\alpha_{2}$ =2.02×10^-2^ ± 1.43×10^-3^(m/V), presence of three components indicate contributions from intrinsic and extrinsic scattering. The quality of fit can be improved by incorporating higher order scattering terms, which is left for future work. The nonlinear Hall conductivity $\sigma_{y}^{2\omega}$ reveals the dominant contribution is scattering intrinsic BCD contributions persistent.

The reproducibility of these results indicates that the nonlinear transport is robust in YbCo_6_Ge_6_, with a similar temperature dependence as shown in SI Fig. 15, where normalised *V_y_^2ω^* is plotted as a function of temperature. The appearance of maxima observed at slightly different temperatures reveals different degrees of disorder in the samples, and is also likely related to the possible transformation to low-symmetry phases associated with lattice distortions ^11, 25^. As the nonlinear Hall effect is highly sensitive to disorder in YbCo_6_Ge_6_, extrinsic effects remain dominant in the observation of NL transport.

**Supplemental Figure 15:** Normalised nonlinear Hall voltage *V_y_^2ω^* in different samples as a function of temperature (T).

(1) Wang, Y.; McCandless, G. T.; Wang, X.; Thanabalasingam, K.; Wu, H.; Bouwmeester, D.; van der Zant, H. S. J.; Ali, M. N.; Chan, J. Y. Electronic Properties and Phase Transition in the Kagome Metal Yb0.5Co3Ge3. *Chemistry of Materials* **2022**, *34* (16), 7337-7343. DOI: 10.1021/acs.chemmater.2c01309.

(2) Meduri, R.; Plata, M. A.; McCandless, G. T.; Schundelmier, B. C.; Ghafoor, M.; Wei, K.; Chan, J. Y. Evolution of Structural Order and Magnetic Anisotropy in Yb0.5(Co1–xFex)3Ge3 through Doping of a Kagome Lattice. *Chemistry of Materials* **2025**, *37* (6), 2302-2313. DOI: 10.1021/acs.chemmater.5c00119.

(3) Weiland, A.; Eddy, L. J.; McCandless, G. T.; Hodovanets, H.; Paglione, J.; Chan, J. Y. Refine Intervention: Characterizing Disordered Yb0.5Co3Ge3. *Crystal Growth & Design* **2020**, *20* (10), 6715-6721. DOI: 10.1021/acs.cgd.0c00865.

(4) Huang, J.; Chen, L.; Huang, Y.; Setty, C.; Gao, B.; Shi, Y.; Liu, Z.; Zhang, Y.; Yilmaz, T.; Vescovo, E.; et al. Non-Fermi liquid behaviour in a correlated flat-band pyrochlore lattice. *Nature Physics* **2024**, *20* (4), 603-609. DOI: 10.1038/s41567-023-02362-3.

(5) Khanenko, P.; Hafner, D.; Semeniuk, K.; Banda, J.; Lühmann, T.; Bärtl, F.; Kotte, T.; Wosnitza, J.; Zwicknagl, G.; Geibel, C.; et al. Origin of the non-Fermi-liquid behavior in ${\mathrm{CeRh}}_{2}{\mathrm{As}}_{2}$. *Physical Review B* **2025**, *111* (4), 045162. DOI: 10.1103/PhysRevB.111.045162.

(6) Das, P.; Saha, P.; Singh, M.; Kumar, P.; Patnaik, S. Quantum linear magnetoresistance and fermi liquid behavior in Kagome Metal Ni3In2S2. *arXiv preprint arXiv:2402.10096* **2024**.

(7) Min, L.; Tan, H.; Xie, Z.; Miao, L.; Zhang, R.; Lee, S. H.; Gopalan, V.; Liu, C.-X.; Alem, N.; Yan, B.; et al. Strong room-temperature bulk nonlinear Hall effect in a spin-valley locked Dirac material. *Nature Communications* **2023**, *14* (1), 364. DOI: 10.1038/s41467-023-35989-0.

(8) Makushko, P.; Kovalev, S.; Zabila, Y.; Ilyakov, I.; Ponomaryov, A.; Arshad, A.; Prajapati, G. L.; de Oliveira, T. V. A. G.; Deinert, J.-C.; Chekhonin, P.; et al. A tunable room-temperature nonlinear Hall effect in elemental bismuth thin films. *Nature Electronics* **2024**, *7* (3), 207-215. DOI: 10.1038/s41928-024-01118-y.

(9) Duan, J.; Jian, Y.; Gao, Y.; Peng, H.; Zhong, J.; Feng, Q.; Mao, J.; Yao, Y. Giant Second-Order Nonlinear Hall Effect in Twisted Bilayer Graphene. *Physical Review Letters* **2022**, *129* (18), 186801. DOI: 10.1103/PhysRevLett.129.186801.

(10) Wang, X.; Hou, T.; Yang, Z.; Li, S.; Jin, T.; Xiao, C.; Sofer, Z.; Qi, D.-C.; Chang, G.; Renshaw Wang, X. Unraveling scattering contributions to the nonlinear Hall effect in topological insulator Bi<sub>2</sub>Te<sub>3</sub>. *Newton* **2026**, *2* (4). DOI: 10.1016/j.newton.2026.100410 (acccessed 2026/07/06).

(11) Tan, H.; Jiang, Y.; McCandless, G. T.; Chan, J. Y.; Yan, B. Three-dimensional higher-order saddle-point-induced flatbands in Co-based kagome metals. *Physical Review Research* **2024**, *6* (4), 043132. DOI: 10.1103/PhysRevResearch.6.043132.

(12) Togo, A.; Chaput, L.; Tadano, T.; Tanaka, I. Implementation strategies in phonopy and phono3py. *Journal of Physics: Condensed Matter* **2023**, *35* (35), 353001. DOI: 10.1088/1361-648X/acd831.

(13) Togo, A. First-principles Phonon Calculations with Phonopy and Phono3py. *Journal of the Physical Society of Japan* **2022**, *92* (1), 012001. DOI: 10.7566/JPSJ.92.012001 (acccessed 2026/07/19).

(14) Ma, T.; Chen, H.; Yananose, K.; Zhou, X.; Wang, L.; Li, R.; Zhu, Z.; Wu, Z.; Xu, Q.-H.; Yu, J.; et al. Growth of bilayer MoTe2 single crystals with strong non-linear Hall effect. *Nature Communications* **2022**, *13* (1), 5465. DOI: 10.1038/s41467-022-33201-3.

(15) Kang, K.; Li, T.; Sohn, E.; Shan, J.; Mak, K. F. Nonlinear anomalous Hall effect in few-layer WTe2. *Nature Materials* **2019**, *18* (4), 324-328. DOI: 10.1038/s41563-019-0294-7.

(16) Qin, M.-S.; Zhu, P.-F.; Ye, X.-G.; Xu, W.-Z.; Song, Z.-H.; Liang, J.; Liu, K.; Liao, Z.-M. Strain Tunable Berry Curvature Dipole, Orbital Magnetization and Nonlinear Hall Effect in WSe2 Monolayer*. *Chinese Physics Letters* **2021**, *38* (1), 017301. DOI: 10.1088/0256-307X/38/1/017301.

(17) He, P.; Isobe, H.; Zhu, D.; Hsu, C.-H.; Fu, L.; Yang, H. Quantum frequency doubling in the topological insulator Bi2Se3. *Nature Communications* **2021**, *12* (1), 698. DOI: 10.1038/s41467-021-20983-1.

(18) Kumar, D.; Hsu, C.-H.; Sharma, R.; Chang, T.-R.; Yu, P.; Wang, J.; Eda, G.; Liang, G.; Yang, H. Room-temperature nonlinear Hall effect and wireless radiofrequency rectification in Weyl semimetal TaIrTe4. *Nature Nanotechnology* **2021**, *16* (4), 421-425. DOI: 10.1038/s41565-020-00839-3.

(19) Liu, S.; Burgos, R.; Zhang, E.; Wang, N.; Qiang, X.-B.; Li, C.; Zhang, Q.; Du, Z. Z.; Zheng, R.; Chen, J.; et al. Room-temperature nonlinear transport and microwave rectification in antiferromagnetic MnBi2Te4 films. *Communications Physics* **2024**, *7* (1), 413. DOI: 10.1038/s42005-024-01897-y.

(20) Cheng, B.; Gao, Y.; Zheng, Z.; Chen, S.; Liu, Z.; Zhang, L.; Zhu, Q.; Li, H.; Li, L.; Zeng, C. Giant nonlinear Hall and wireless rectification effects at room temperature in the elemental semiconductor tellurium. *Nature Communications* **2024**, *15* (1), 5513. DOI: 10.1038/s41467-024-49706-y.

(21) Orlova, N. N.; Timonina, A. V.; Kolesnikov, N. N.; Deviatov, E. V. Gate-Dependent Nonlinear Hall Effect at Room Temperature in Topological Semimetal GeTe. *Chinese Physics Letters* **2023**, *40* (7), 077302. DOI: 10.1088/0256-307X/40/7/077302.

(22) Min, L.; Zhang, Y.; Xie, Z.; Ayyagari, S. V. G.; Miao, L.; Onishi, Y.; Lee, S. H.; Wang, Y.; Alem, N.; Fu, L.; et al. Colossal room-temperature non-reciprocal Hall effect. *Nature Materials* **2024**, *23* (12), 1671-1677. DOI: 10.1038/s41563-024-02015-7.

(23) Lee, S. H.; Iwaya, T.; Nakayama, K.; Lim, T. Y.; Min, L.; He, J.; Wang, Y.; Gopalan, V.; Xie, Z.; Pan, X.-C.; et al. Disorder Scattering Induced Large Room Temperature Nonlinear Anomalous Hall Effect in a Semiconductor CdGeAs2. *Advanced Materials* **2026**, *38* (7), e14217. DOI: <https://doi.org/10.1002/adma.202514217>.

(24) Kumar, D.; Sharma, R.; Wang, F.; Liu, Y.; Zhao, S.; Yang, H. Quantum Rectification Based on Room Temperature Multidirectional Nonlinearity in Bi2Te3. *Nano Letters* **2024**, *24* (40), 12545-12551. DOI: 10.1021/acs.nanolett.4c03517.

(25) Korshunov, A.; Fuller, C. A.; Lim, C. Y.; Kar, A.; Roychowdhury, S.; Chernyshov, D.; Shekhar, C.; Bosak, A.; Felser, C.; Blanco-Canosa, S. Disentangling structural order and disorder in kagome ${\mathrm{Yb}}_{0.5}{\mathrm{Co}}_{3}{\mathrm{Ge}}_{3}$. *Physical Review B* **2025**, *112* (17), 174103. DOI: 10.1103/rs1h-njky.
